# Misleading Goldmann applanation tonometry in a post-LASIK eye with interface fluid syndrome

**DOI:** 10.4103/0301-4738.64133

**Published:** 2010

**Authors:** Sirisha Senthil, Varsha Rathi, Chandrasekhar Garudadri

**Affiliations:** LV Prasad Eye Institute, LV Prasad Marg, Banjara Hills, Hyderabad-500034, India

**Keywords:** Interface fluid, LASIK, misleading tonometry, optical coherence tomography

## Abstract

A 21-year-old myope presented with decreased vision and corneal edema following vitreoretinal surgery for retinal detachment. While intraocular pressure (IOP) measurement with Goldmann applanation tonometer (GAT) was low, the digital tonometry indicated raised pressures. An interface fluid syndrome (IFS) was suspected and confirmed by clinical exam and optical coherence tomography. A tonopen used to measure IOP through the peripheral cornea revealed elevated IOP which was the cause of the interface fluid. Treatment with IOP-lowering agents resulted in complete resolution of the interface fluid. This case is being reported to highlight the fact that IFS should be suspected when there is LASIK flap edema and IOP readings using GAT are low and that GAT is not an optimal method to measure IOP in this condition. Alternative methods like tonopen or Schiotz tonometry can be used.

Fluid collection in the flap interface, called interface fluid syndrome (IFS) is a rare complication following laser-assisted *in situ* keratomileusis (LASIK) surgery.[1] It has been reported secondary to raised intraocular pressure (IOP), endothelial decompensation and uveitis.[2] Measuring IOP in post-LASIK eyes with IFS using Goldmann applanation tonometer (GAT) can result in erroneous IOP readings. Use of tonopen to record IOP in the peripheral cornea is reported to be more accurate in these eyes.[3] To our knowledge there is no report on IFS due to raised IOP in a post-LASIK eye following a vitreoretinal surgery. High-resolution optical coherence tomography (OCT) is helpful in confirming the diagnosis of IFS and to identify the underlying pathology.[4] Finding out the cause of IFS and appropriate management helps in complete resolution of IFS and restoration of vision.[3] The aim of this report is to highlight the fact that using GAT in post-LASIK eyes with IFS can result in underestimation of IOP.

## Case Report

A 21-year-old male presented with complaints of sudden painless decrease in vision in the left eye (LE) since one month. Two years earlier, he had undergone LASIK in both eyes for a myopic refractive error. On examination right eye (RE) was normal and LE showed a subtotal retinal detachment with proliferative vitreoretinopathy Grade B. He underwent belt buckling with pars plana vitrectomy and silicone oil injection in the LE. Following an uneventful surgery and postoperative course, his vision was 20/25 in RE and 20/80 in LE; IOP was 12 mmHg in both the eyes.

Three months after he underwent emulsified silicone oil removal, he presented with decreased vision in the LE. His vision was 20/25 in RE and counting fingers at 1½ meter in LE. IOP was 17 mmHg in RE and 2 mmHg in LE with GAT. However, the digital IOP was high in the LE. IOP measured with the tonopen (Medtronic Ophthalmic, Jacksonville, FL) in the peripheral cornea was 16 in RE and 30 mmHg in LE. The LASIK flap was well opposed in RE; there was flap edema in the LE with a clear space between the flap and the stromal bed [Fig. 1a]. An IFS was suspected and confirmed using high-resolution OCT [Fig. 1b]. The flap thickness was 211 μ, interface fluid pocket was 206 μ and residual bed was 279 μ. The endothelial count was 2788 cells/mm^2^ in RE and 2866 cells/mm^2^ in LE. Optic disc examination revealed 0.4:1 and 0.7:1 cupping in the RE and LE respectively with thinning of superior rim in the LE. He was started on topical timolol maleate 0.5% twice daily, brimonidine 0.15% thrice daily, travoprost 0.004% once at bedtime, for the LE along with tab. acetazolamide 250 mg three times per day for two days followed by 125 mg twice a day for three weeks. Over a three-month period, vision remained stable in both eyes, the IOP measured by GAT and tonopen was similar for the RE, but was between 3-23 mm Hg with GAT and 25-34 mm Hg with the tonopen in the LE. By two months, with medical control of IOP with topical beta-blockers, prostaglandin analogues, alpha agonist and systemic carbonic anhidrase inhibitors, the interface fluid had disappeared completely as confirmed by OCT [Fig. 2a, b], IOP was 17 mm Hg with GAT and 19 with tonopen; and the vision was finger counting at 1½ meter in the LE. After resolution of the IFS, the flap thickness was 159 μ and residual bed was 292 μ.

**Figure 1a:**
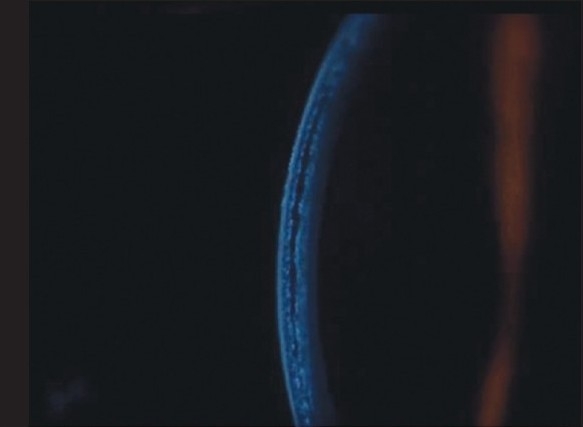
Slit view of the cornea showing interface fluid

**Figure 1b:**
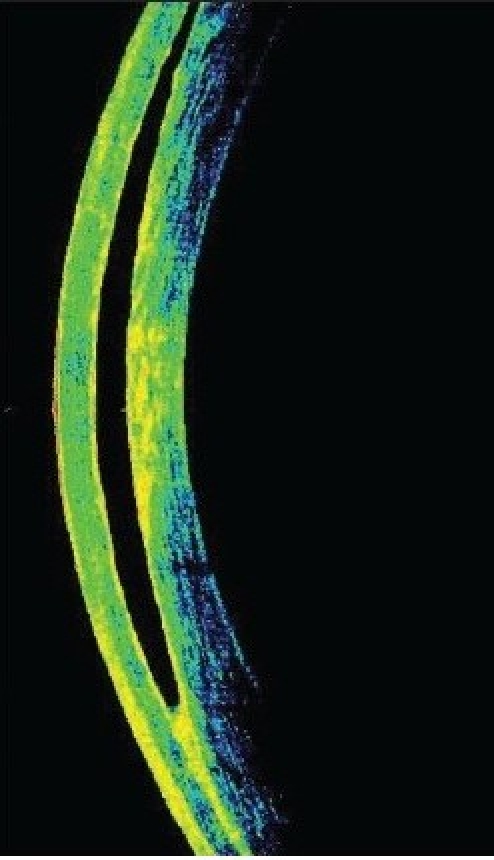
Anterior segment OCT showing interface fluid as optically empty space in the flap-stromal interface

**Figure 2a:**
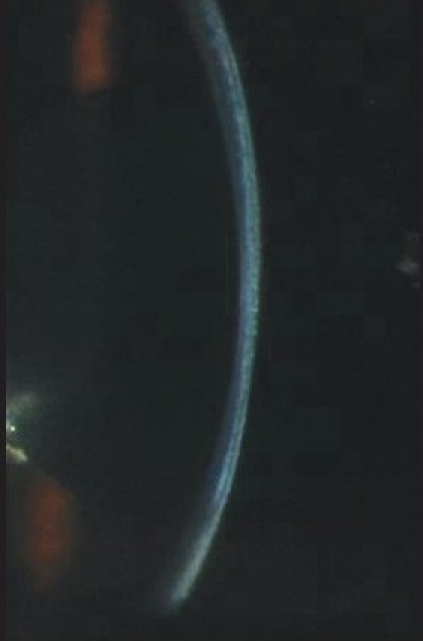
Slit view of the cornea with resolved interface fluid with well apposed flaps

**Figure 2b:**
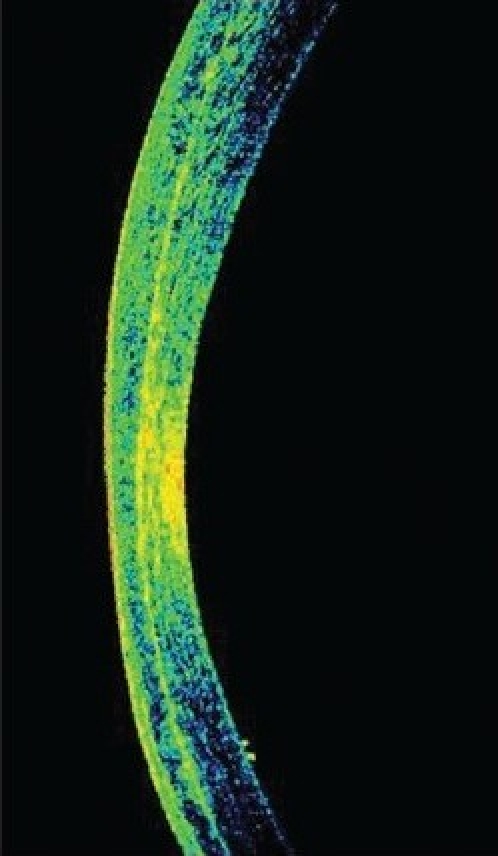
Anterior segment OCT showing no separation of the interface

## Discussion

LASIK surgery for myopia is a common refractive surgical procedure. Rise in IOP in post-LASIK eyes has been reported secondary to the topical corticosteroids used in the postoperative period.[5] In post-LASIK eyes, fluid collecting between stromal bed and the flap interface is described as IFS and is usually secondary to raised IOP. In this patient, the rise in IOP was probably secondary to emulsified silicone oil which was used as a tamponade during retinal detachment (RD) surgery. Interface fluid accumulation and posterior stromal edema after RD surgery has been reported due to transient endothelial decompensation in the immediate postoperative period.[6]

Clinically, severe IFS can be detected with a careful slit-lamp examination. IFS appears as an optically empty space between the flap and the residual stromal bed. However, early interface changes associated with IFS could masquerade as diffuse lamellar keratitis (DLK).[7] Treatment for DLK is frequent steroids. IFS can be associated with epithelial ingrowth. The epithelial cells in the interface could be the source of the fluid. Surgical removal of the epithelial ingrowth, combined with medical therapy, is necessary to resolve the interface fluid. Temporary or permanent corneal endothelial cell dysfunction can lead to IFS.[6] When the corneal endothelium is damaged, aqueous humor typically diffuses into the corneal stroma resulting in corneal swelling predominantly in the posterior two-thirds of the corneal stroma, and diffusion of fluid into the interface creates a fluid pocket in the lamellar interface. The high-resolution images provided by the cornea anterior segment module of the Fourier domain OCT can be useful in visualizing the exact location, extent and height of fluid collection, epithelial ingrowth and noncellular reflective deposits which cannot be differentiated clinically. It is also useful for follow-up to check for resolution of the fluid.

Corneal properties like central corneal thickness (CCT), corneal hydration and corneal curvature can influence GAT readings. Cases with severe IFS are often associated with falsely low central GAT readings and the IOP measured may represent the pressure of the fluid-filled pocket rather than the true IOP.[8] In post-photorefractive keratectomy and LASIK eyes, IOP measured using tonopen is reported to be more accurate than with GAT.[9] In our case, due to IFS there was underestimation of IOP by GAT, but the tonopen recordings in the peripheral cornea were higher. To our knowledge there is no tonometer that gives accurate readings in such situations. However, in this condition, we want to know the approximate IOP range which helps us to treat the condition. In situations like ours, tonopen, Schiotz tonometer or scleral tonometers which are less likely to be influenced by corneal rigidity may be better options to measure IOP. Role of newer tonometers like ocular response analyzer (which is less likely to be affected by corneal properties) in post-LASIK eyes with IFS is not known.

With the increasing use of LASIK, clinicians are more likely to encounter a clinical situation like ours. Frequency of steroid responsiveness is much higher in myopes. In these people in the post-LASIK period, limiting the instillation of topical corticosteroids and measuring the IOP after routine LASIK surgery are probably the easiest and most important steps to prevent IFS.

A high index of suspicion is important in making a diagnosis of IFS. IFS should be suspected when there is LASIK flap edema and IOP readings using GAT are low. Lack of awareness that GAT readings can lead to erroneously low IOP measurements in eyes with IFS can lead to misdiagnosis and inappropriate treatments resulting in irreversible loss of vision.
